# A cross sectional study to determine the prevalence of cough and its impact in patients with lung cancer: a patient unmet need

**DOI:** 10.1186/s12885-019-6451-1

**Published:** 2020-01-06

**Authors:** Amélie Harle, Alex Molassiotis, Oliver Buffin, Jack Burnham, Jaclyn Smith, Janelle Yorke, Fiona H. Blackhall

**Affiliations:** 1 Dorset Cancer Centre, Poole NHS Foundation Trust, Longfleet Road, Poole, BH15 2LB UK; 2 Department of Medical Oncology, The Christie NHS Foundation Trust, Manchester, UK; 3 School of Nursing, The Hong Kong Polytechnic University, Hong Kong SAR, China; 4 School of Health Sciences, Faculty of Biology, Medicine and Health, University of Manchester, Manchester, UK; 5 Royal Gwent Hospital, Wales Deanery, Newport, Gwent, Wales, UK; 6 Department of General Medicine, Gloucestershire Hospitals NHS Foundation Trust, Cheltenham, UK; 7 Division of Infection, Immunity and Respiratory Medicine, University of Manchester, Manchester, UK; 8 Manchester University NHS Foundation Trust, Manchester, UK; 9 Christie Patient Centred Research, The Christie NHS Foundation Trust, Manchester, UK; 10 Division of Cancer Sciences, University of Manchester, Manchester, UK

**Keywords:** Cough, Lung cancer, Prevalence, Impact, Quality of life

## Abstract

**Background:**

There is absence of literature related to cough prevalence and its characteristics in lung cancer patients, with information deriving only from broader symptoms occurrence studies. The aims of this study were to provide a snapshot of the prevalence of all-cause-cough in lung cancer patients and to characterise cough in terms of its impact and severity.

**Methods:**

A cross-sectional study recruiting consecutive lung cancer patients over a pre-defined period of time and using cough-specific validated tools in a tertiary referral centre in the UK, including a cough severity VAS and the Manchester Cough in Lung Cancer scale (MCLCS).

**Results:**

Data was collected from 202 patients. All-cause cough prevalence was 57% (through VAS) both in the screened (*N* = 223) and research (*N* = 202) population or 67% (through the MCLCS), and cough severity was moderate at a mean of 32 mm (in a 100 mm VAS). Age, sex, smoking status, lung cancer histology, stage and comorbidities were not associated with cough prevalence. The only variable associated with lower cough reports was being ‘on anticancer treatment’; fewer patients on treatment reported a cough (40%) compared to those off treatment (54%) (*p* = 0.04). The impact of cough (as measured by MCLCS) was also significant (mean score = 22). About 18% of patients felt moderate/severe distress from their cough and about 15% often or always reported disturbed sleep due to coughing. Half the patients felt their cough warranted treatment.

**Conclusions:**

Cough is a common symptom in lung cancer with considerable impact on patients’ lives. Cough presence and severity should regularly be assessed in clinical practice. There is an urgent need to focus on developing more potent antitussive treatments and improve the management of this complex and distressing symptom.

## Background

Lung cancer-related cough is an important unmet clinical need for which morbidity and distress are often underestimated by health professionals [1, 2]. Cough impacts on physical, psychological and social aspects of daily living [2, 3], contributes to pain, fatigue, insomnia and dyspnoea [2], increases anxiety in patients and carers [4] and leads to social isolation [2]. Whilst it is recognised that cough is a common symptom in patients with lung cancer, cough prevalence rates vary significantly in the literature. This may be explained by the differing methodologies and patient groups used in these trials, with different comorbidities and environmental factors. Cough may have multiple causes, even within a lung cancer population. Patients with lung cancer can suffer from acute causes of cough such as an infection, chronic causes of cough due to co-morbidities such as Chronic Obstructive Pulmonary Disease (COPD) or smoking and/or may have a cough due to the effects of the malignancy and its treatment. Measurement of cough may also be an issue, as most studies in the past have not used cough-specific assessments, obtained cough data from items in quality of life scales or have used generic cough instruments [5–7] that may not provide reliable indications of cough in the lung cancer context.

There is minimal work with lung cancer patients to date focusing on cough specifically, as almost all information to date derives from wider symptom burden studies. A report of two lung cancer cohorts, one in 2002 (*n* = 108) and one in 2012 (*n* = 100) showed that severe symptom rates were similar over the decade, and cough, alongside fatigue and depression were the most common symptoms experienced [6]. Studies assessing symptom prevalence in lung cancer patients show that cough is a common bothersome symptom. In a US study with over 400 patients, data suggested that about 80% of patients reported a cough, with 64.8% of the study population reporting a persistent cough [8]. Another study from the same authors, based in France and Germany in over 800 patients reported a cough prevalence rate of 93% [9]. Both studies only included patients with non-small cell lung cancer (NSCLC) histology, advanced stage disease (Stage IIIB/IV) and patients receiving chemotherapy (1st, 2nd or 3rd line). In another study, Tishelman et al. describes the longitudinal variation in symptom prevalence, intensity and distress in a cohort of 400 patients with lung cancer, using a quality of life scale at six time-points during the 1st year after diagnosis [3]. This study reported a cough prevalence of 70% at the time of lung cancer diagnosis and 81% in the month prior to death [3].

Other studies show cough prevalence rates of 64.1% preoperatively and 59.9% 5 months later using the Memorial Symptom Assessment Scale [5], or moderate/severe cough of 39.6% of early stage patients and 44.5% in late stage patients, using a non-validated symptom scale [7]. In a large cohort of 447 lung cancer patients, declining quality of life was linked with five symptoms, including cough, and the symptom burden was the same even among those patients whose quality of life improved [10]. In the only observational study having cough as its primary focus (*n* = 177 at entry and 153 at 60 days assessment), higher cough severity at study entry was associated with female sex, asthma, and reflux disease, whereas cancer stage, cancer histology, smoking or chronic obstructive pulmonary disease (COPD) were not associated with cough severity or cough impact [11].

The latter study also showed that cough is a frequent and distressing symptom and an unmet clinical need [11]. It demonstrated that patients with lung cancer suffer from a severe and frequent cough; worse than in patients with COPD and asthma and as severe and frequent as in patients who present to specialist chronic cough clinics with cough as their presenting symptom [11]. The objectives of this study were to determine the prevalence of cough in a cohort of patients with lung cancer and to characterise the cough in terms of its impact and severity using validated cough assessment tools. It did not seek to determine the underlying cause of the cough but rather describe prevalence, severity and impact in a clinic population.

## Methods

### Study design

This was a cross-sectional study using consecutive patients attending lung cancer oncology outpatient clinics in a referral centre in the Northwest of England between 13th June 2013 and 14th May 2013.

#### Participants

To minimise bias, consecutive patients were approached by their healthcare team during a predefined 5 week period. All patients were asked whether they had a cough (‘yes’/‘no’ response). Cough was not formally defined. It was felt that it is a term that is easily understood by patients. Demographic, cancer and cancer treatment data were collected on all patients. If patients consented to the cross-sectional study, they were asked additional questions about the presence of reflux symptoms. If they had reported a cough, they were asked further about their cough by the researchers: “Is your cough painful?” and “Do you feel that your cough warrants treatment?” Patients subsequently completed the Manchester Cough in Lung Cancer scale [12] and the cough severity visual analogue scale on the same day. Patients were eligible to participate if they had a diagnosis of lung cancer (NSCLC or SCLC), were fit enough and able to read and respond to questions in English. Ethical approval for the study was obtained from North East-Tyne & Wear South Research Ethics Committee: Approval Number 13/NE/0066.

#### Assessments

Once enrolled on the study, patients were asked by researchers: “Is your cough painful?” and “Do you feel that your cough warrants treatment?” Both questions had “yes/no” answers. The timeframe for all assessments was during the past week.

Then, cough severity was measured using a 100 mm visual analogue scale, where the start of the line is defined as “no cough” and the end of the line is defined as “worse cough severity”. Patients were asked to show the severity of their cough by marking the line at the point which they felt most represented the severity of their cough. Although not formally validated, this tool is a widely accepted in the field of cough research. It is responsive to change and clinically meaningful [13].

Manchester Cough in Lung Cancer scale (MCLCS): This is a 10-item lung cancer-cough specific quality of life scale measuring the impact of cough on patients’ life. This validated scale reported a Cronbach alpha of 0.86 and high test-retest reliability [12].

The clinical factors identified as being potentially associated with the presence of cough were: time from diagnosis, age, sex, smoking (never vs current/ex), stage (early vs late), histology (SCLC vs NSCLC), self-reported co-morbidities (asthma, COPD, GORD) and ECOG performance status.

#### Statistical analysis

Since this study was primarily a prevalence study, there was no pre-defined ceiling to the number of patients enrolled. Statistical analyses were performed with the use of SPSS software, version 19.0. Descriptive statistics were used to estimate the frequencies, means, and standard deviations of the study variables. Non-parametric tests were used to compare differences between cough rates and personal or clinical characteristics.

## Results

### Study recruitment

**Fig. 1:**
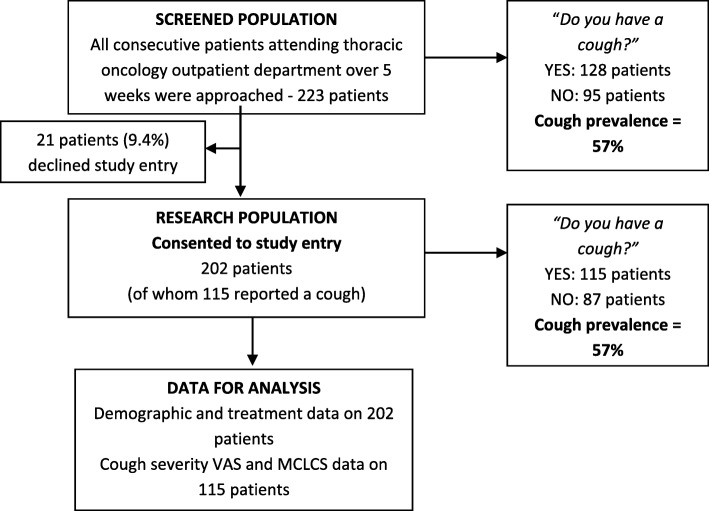
Flowchart of the participant recruitment to the study

All consecutive patients attending the thoracic oncology outpatient clinics of the study hospital were recruited over 5 weeks. A total of 223 patients were screened. Of these, 90.6% (*n* = 202) consented to participate in the study (Fig. 1). The cough prevalence in the screened population was 57%, which was identical to the prevalence in the research population. No variable had more than 1% missing data, with absolute numbers ranging from 199 to 202 for each demographic, cancer, treatment or cough variable assessed.

### Sample characteristics

The sample’s mean age was 66 years (SD = 8.93). Just over half (53%) patients, were male. The majority had a history of smoking and the median number of pack years on ever smokers was 36.8 (25th–75th IQR 17.5–49.7). With respect to co-morbidities, 75 (37%) patients reported nausea and 106 (53%) patients reported gastro-oesophageal reflux symptoms. Less than half the study population was on anticancer therapy (*n* = 91, 46%). Of these patients, the vast majority were on palliative intent treatment - 81 patients (89%). Of the patients who were not receiving anticancer therapy, the majority (42 patients, 38%) were on follow-up following palliative treatment, and 31 patients (28%) were newly diagnosed and at pre-treatment. Other sample characteristics can be seen in Table 1.

**Table 1:** Sample characteristics (*n* = 202)

	*N* (%)
Performance status	
0	27 (13)
1	72 (36)
2	71 (35)
3	32 (16)
Lung cancer histology	
Non-small lung cancer (NSCLC)	135 (67)
Small cell lung cancer (SCLC)	63 (31)
Mixed histology	3 (2)
Stage	
Early stage NSCLC	26 (13)
Stage IIIb-IV NSCLC	110 (55)
Early/limited stage SCLC	17 (9)
Extensive stage SCLC	46 (23)
Smoking status	
Ex-smoker	135 (67)
Current smoker	47 (23)
Never smoked	19 (10)

### A comparison of clinical characteristics between patients with and without cough

Any patient who reported the presence of a cough at trial entry was assumed to have a cough, irrespective of its cause, severity or impact. All other patients were defined as having no cough. Overall, 115/202 patients reported a cough; therefore the cough prevalence rate was 57%.

Patient baseline demographic and cancer characteristics such as age, sex, smoking history, performance status, stage of cancer, histology, NSCLC histological subtype, cancer treatment intention, cancer treatment type and reasons for not receiving cancer treatment did not differ significantly between the two groups. The only variable that differed significantly between the two groups was the proportion of patients receiving anticancer therapy (Table 2). However, there was a non-significant trend (*p* = 0.09) for a greater proportion of patients with a worse PS 2–3 reporting a cough compared to those with a better PS 0–1: 58% vs 42%. (58%). This was also the case with those with advanced disease, NSCLC and adenocarcinomas.

**Table 2:** Comparison of clinical characteristics between patients with and without a cough

Characteristic	Subgroup	Cough, *N* (%)	No Cough, *N* (%)	*p*-value
Mean age in years (SD)		67 years (9.02)	66 years (8.85)	0.56
Male sex		57 (50)	49 (56)	0.34
Smoking status	Never smoked	12 (10)	7 (8)	0.44
	Former	73 (64)	62 (72)	
	Current	30 (26)	17 (20)	
Median no. pack years on ever smokers (25th–75th IQR)		38 (17.5–46)	33 (17.5–50)	1.00
Performance status	0	15 (13)	12 (14)	0.09
	1	33 (29)	39 (45)	
	2	46 (40)	25 (29)	
	3	21 (18)	11 (12)	
Stage	Early NSCLC (incl IIIA)	14 (12)	12 (14)	0.61
	Advanced NSCLC	63 (56)	47 (55)	
	Limited stage SCLC	12 (11)	5 (6)	
	Extensive stage SCLC	24 (21)	22 (25)	
Histology	Non-small cell lung cancer	76 (67)	59 (68)	1.00
	Small cell lung cancer	36 (31)	27 (31)	
	Mixed	2 (2)	1 (1)	
Non-small cell lung cancer histological subtype	Adenocarcinoma	44 (55)	40 (65)	0.29
	Squamous	25 (31)	16 (26)	
	Not otherwise specified	10 (13)	3 (5)	
	Mixed	1 (1)	1 (2)	
	Large cell	0 (0)	1 (2)	
On anticancer therapy	Yes	45 (40)	47 (54)	0.04
What type of anticancer therapy	Chemotherapy	28 (62)	30 (65)	0.44
	Radiotherapy	3 (7)	0 (0)	
	Concurrent	2 (4)	2 (4)	
	Tyrosine-kinase inhibitors	12 (27)	14 (31)	
If not on anticancer therapy, why not	Pre-treatment	16 (23)	13 (31)	0.33
	Post palliative treatment	24 (34)	18 (43)	
	Post curative treatment	6 (9)	3 (7)	
	No further treatment	24 (34)	8 (19)	
Comorbidities	Nausea	42 (36)	33 (38)	0.79
	Gastro-oesophageal reflux	62 (54)	44 (51)	0.70

*NSCLC* Non-small cell lung cancer, SCLC: Small cell lung cancer

### Characteristics and impact of cough in the study population

Half the patients who reported cough felt that their cough warranted treatment and one-quarter of them reported their cough to be painful. The median VAS score showed that most patients graded their cough at moderate level (32 mm, 25th–75th IQR 20–51) whilst the median MCLCS score showed a moderate cough impact score of 22 (25th–75th IQR 16–27) (Table 3). MCLCS data also showed that 39% of patients reported moderate to severe cough; 18% reported significant distress from cough (‘often’, ‘very often’, ‘all of the time’; mean = 1.85/5, SD = 1.14) and 15% reported significant sleep disturbance because of cough (Table 3). Significant correlations were observed between MCLCS cough severity and VAS cough severity (r_s_ = 0.69, *p* < 0.001), MCLCS cough severity and MCLC scale’s cough frequency (r_s_ = 0.54, p < 0.001), and VAS cough severity and MCLCS cough frequency (r_s_ = 0.57, p < 0.001).

**Table 3:** Cough characteristics in the study population

Characteristic	Cough	Comment
Is cough painful? ‘yes’, N (%) from those reporting cough	26 (23)	
Does cough warrant treatment? ‘yes’, N (%) from those reporting cough	60 (52)	
Is Cough Painful? Median cough severity VAS score for those experiencing cough (25^th^-75^th^ IQR)	32mm (20-51)	Higher score = worse cough severity (100mm scale)
Median MCLCS score for those experiencing cough (25^th^-75^th^ IQR)	22 (16-27)	Higher score = worse cough impact; maximum possible score=40
	*N* (%)	
Cough reported	115 (57)	Initial assessment (n=223)
Cough reported	135 (67)	MCLCS data
Difficulty breathing because of cough^a^	26 (19)	MCLCS data, (‘often’, ‘very often’, ‘all of the time’)
Disturbed sleep because of cough^a^	20 (15)	MCLCS data, (‘often’, ‘very often’, ‘all of the time’)
Distress from cough^a^	25 (18)	MCLCS data, (‘often’, ‘very often’, ‘all of the time’)
Frustration because of cough^a^	40 (29)	MCLCS data, (‘often’, ‘very often’, ‘all of the time’)
Cough interrupts conversations^a^	32 (23)	MCLCS data, (‘often’, ‘very often’, ‘all of the time’)
In control of your cough^a^	69 (50)	MCLCS data, (not at all; some of the time)
Worry that cough means your condition is getting worse^a^	43 (31)	MCLCS data, (‘often’, ‘very often’, ‘all of the time’)

*MCLCS* Manchester Cough in Lung Cancer Scale, *VAS* Visual Analogue Scale, *IQR* Interquartile range

^a^Only for those reporting cough

## Discussion

The data presented provide a “snapshot” of cough prevalence in a “real-life” UK outpatient medical oncology clinic population. Over half of the patients with lung cancer in the current study suffered from a cough; with over half of these feeling that their cough warrants treatment and a quarter reporting a painful cough. Since consecutive patients were approached, the potential for selection bias was minimised. This is supported by the finding that the prevalence was identical between the screened and research populations.

Since our study did not select patients according to stage, histology or cancer therapy, its cough prevalence figure is likely to be more representative of the general lung cancer outpatient population in our hospital compared to the studies by Iyer et al. [8, 9]. The higher cough prevalence rate in past studies [8, 9] may reflect a significant number of patients with a very mild cough, a selection outcome of the assessment methods used. However, other studies show similar rates of cough prevalence with our findings [5, 7, 10]. Furthermore, over 50% of our patients had a performance status of 2–3, whilst only 23% of patients in the European study by Iyer [9] had a performance status > 1. In our study, there was a trend (*p* = 0.09) suggesting that patients with a poorer performance status were more likely to report a cough compared to patients with a performance status of 0–1. Performance status has been shown to be associated with both cough severity and cough impact in patients with lung cancer [11]. The “trend” for its association with cough prevalence is therefore noteworthy. Performance status has previously been shown to be a predictor of symptom burden and quality of life in lung cancer [8, 9]. The prognosis is often shorter in patients with a poor performance status compared to patients with a better performance status score [14, 15] and hence optimising their quality of life during their remaining life-time is of critical importance if we are to maximise their well-being and potentially their overall survival [14, 15] .

Our study found that the only clinical factor associated with cough prevalence was “being on anticancer therapy”. Those patients who were on treatment were less likely to have a cough than patients who were not receiving treatment (40% vs 54%, *p* = 0.04). Interestingly, the cough prevalence rates in both studies by Iyer [8, 9] were high despite the fact that all patients were receiving chemotherapy. It is likely that factors other than being on anticancer treatment also predict the prevalence of cough in lung cancer, such as sex, tumour location, use of opioids [11], and may explain the differences in cough prevalence rates between studies. Furthermore, in another study, chemotherapy was not associated with lower cough [5]. These add weight to the argument that anticancer treatments may not fully manage cough and effective antitussives are required for the lung cancer population.

It is noteworthy that commonly held assumptions about clinical factors associated with cough such as a history of smoking, comorbidities such as COPD or cancer characteristics such as tumour location, or the type of histology were not found to be associated with cough prevalence in this study. This is surprising but demonstrates that cough remains poorly understood. This is an area that requires more focus in future research using larger samples, as currently the available evidence on which to make comparisons with our findings is almost non-existent.

The mean VAS cough severity score was moderate to mild (32 mm) in our study, similar to past symptom studies [5] and to the more recently published longitudinal study [11]. However, this score is higher than reported series of patients with asthma and COPD [16, 17] and in keeping with patients with chronic cough presenting to specialist cough clinics [18].

Our data demonstrate that cough was associated with a significant impact on physical, psychological and social aspects of daily life. In the original MCLC scale development study [12], the mean total MCLCS score in 139 patients with different histologies of lung cancer including mesothelioma was 18.3 (range 1–39). Furthermore, a longitudinal study of 177 patients with lung cancer reported a mean MCLCS score of 24 [11], similar to the current study. Hence, all three studies providing MCLCS data show a moderate impact of cough on aspects of life. Whilst there is an association between cough and quality of life, the poorer quality of life is not necessarily caused by the cough. This is an observational study and therefore causality cannot be attributed. Nevertheless, lung cancer patient-reported experiences of cough highlight its major impact on socializing, psychological status, embarrassment [2]. However, it is acknowledged that cough is rarely a sole symptom in lung cancer. It may be that it is the combination of symptoms that may be more strongly impacting on a patient’s quality of life.

Several publications describe the consequences of cough that include physical symptoms such as pain, psychological symptoms such as anxiety and social implications such as no longer going out to restaurants [2, 4, 19]. Therefore, with lung cancer-related cough severity scores at the moderate level reported in this study, it is not surprising that many patients with lung cancer (52%) felt that their cough warrants treatment. This is similar to another study where 62% of patients with lung cancer felt their cough warrants treatment [11]. A significant proportion of patients, with lung cancer pathology that often causes chest pain and rib pain, report a painful cough since the sudden and sometimes forceful nature of a cough is likely to exacerbate this pain. Since cough is an intermittent symptom, it is difficult to predict use of analgesia to provide adequate pain relief in patients who suffer from a painful cough. The approach to such patients may be to improve their cough rather than to treat the pain relating to the cough specifically.

Understanding what constitutes a severe cough is complex and key to this is the appropriate selection of tools to generate robust data. A study in chronic cough patients elegantly demonstrated that cough severity had three domains: intensity, disruptiveness and frequency [20]. Therefore no single subjective or objective value is sufficient to fully characterise cough severity. The longitudinal assessment of cough is also warranted if we are to better understand its variation and predictors as lung cancer progresses. A pragmatic approach for a predefined question asked at a defined time point on the disease trajectory may be an acceptable method to determine cough prevalence.

While the consecutive patient sampling and the number of participants are strengths of the study, limitations of the study include the cross-sectional design and the single-centre study. Since we wanted to capture a snapshot of cough in patients with lung cancer attending outpatient clinics, duration of cough was not measured; hence, incidental or transient cough may have inflated the prevalence of cough in our sample, and this needs attention in future research.

Cough was not formally defined at the outset in this study. Any cough, whether incidental, transient or chronic, relating to a comorbidity or the cancer would have been captured in this study. This study therefore reports the prevalence of “all-cause” cough. The study researchers felt that to seek to define cough more precisely by either using a time-frame or attributing a cause to the cough such as “COPD or Lung Cancer” could lead to significant reporting biases. As our understanding of cough in lung cancer increases, this is an area that warrants further research.

A future study should assess the causes of cough, how much this cough is attributable to cancer or non-cancer disease or whether its cause is treatable (e.g. infection, asthma, heart failure), non-treatable (e.g. lymphagitis or tracheal invasion of cancer) or whether it is self-limiting (e.g. viral infection). While this information will be vital for a therapeutic study, our study however has established the prevalence of all-cause-cough in patients with lung cancer attending regular clinics in a regional centre and shows the proportion of patients that need to be treated and the extent of the problem.

It was unknown whether any of these patients had received (prescribed or over-the-counter) antitussives or other medication that may impact on cough (e.g. opioids, steroids, bronchodilators or angiotensin-converting-enzyme inhibitors) at the time of study entry. Information on history of asthma or COPD was not collected. However, of note, in a previously published longitudinal study these were not shown to relate to cough severity or impact in a lung cancer population [11].

Finally, future research could explore associations of cough with site of disease (e.g. central/proximal vs peripheral disease), as site of disease may be a factor in cough prevalence. The above also show how complex it is to assess cancer-related cough (vs any cough) in patients with lung cancer and that often multiple causes may contribute to the development of cough in lung cancer; this calls for different treatment approaches to manage cough adequately [21]. Future research on the underlying mechanisms and causes of cough may further identify potential novel therapies.

## Conclusions

This study is one of the first to use validated lung-cancer specific cough assessment tools in a real-world population of patients attending lung cancer clinics and demonstrates that cough is a common symptom, affecting more than half of patients in this study and associated with considerable impact on the patients’ life. In the absence of effective antitussive therapies, cough remains an unmet need for these patients. The evidence-base for antitussive treatments for lung cancer is minimal and of poor quality, summarised in a Cochrane review [22] and a guideline by the American College of Chest Physicians [21]. For optimal cough outcomes, there is an urgent need for more attention and investment in identifying causes of cough in the lung cancer population, its potential underlying mechanisms and to test new antitussive treatments. Research into the impact of the cough on a patient is crucial. Lack of recognition of this common, distressing symptom, means that it remains an unmet need.

## Data Availability

The datasets used and/or analysed during the current study are available from the corresponding author on reasonable request.

## Abbreviations

COPD: (Chronic Obstructive Pulmonary Disease)
ECOG: (Eastern Cooperative Oncology Group)
IQR: (Interquartile range)
MCLCS: (Manchester Cough in Lung Cancer Scale)
NSCLC: (Non Small Cell Lung Cancer)
PS: (Performance Status)
SCLC: (Small Cell Lung Cancer)
VAS: (Visual Analogue Scale),
